# Autosomal dominant optic atrophy and cataract “plus” phenotype including axonal neuropathy

**DOI:** 10.1212/NXG.0000000000000322

**Published:** 2019-04-01

**Authors:** Alejandro Horga, Enrico Bugiardini, Andreea Manole, Fion Bremner, Zane Jaunmuktane, Lois Dankwa, Adriana P. Rebelo, Catherine E. Woodward, Iain P. Hargreaves, Andrea Cortese, Alan M. Pittman, Sebastian Brandner, James M. Polke, Robert D.S. Pitceathly, Stephan Züchner, Michael G. Hanna, Steven S. Scherer, Henry Houlden, Mary M. Reilly

**Affiliations:** From the Department of Neuromuscular Diseases (A.H., A.C., M.G.H., M.M.R.), UCL Queen Square Institute of Neurology and the National Hospital for Neurology and Neurosurgery, University College London Hospitals; Department of Molecular Neuroscience (A.M.P., H.H.), UCL Queen Square Institute of Neurology; Department of Neuro-ophthalmology (F.B.F.R.C.O.), the National Hospital for Neurology and Neurosurgery, University College London Hospitals; Division of Neuropathology (Z.J., S.B.), the National Hospital for Neurology and Neurosurgery, University College London Hospitals; Department of Clinical and Movement Neurosciences (Z.J.), UCL Queen Square Institute of Neurology, London, United Kingdom; Department of Neurology (L.D., S.S.S.), Perelman School of Medicine, University of Pennsylvania, Philadelphia; Department of Human Genetics and Hussman Institute for Human Genomics (A.P.R., S.Z.), University of Miami, FL; Department of Neurogenetics (C.E.W., J.M.P.), the National Hospital for Neurology and Neurosurgery, University College London Hospitals; Neurometabolic Unit (I.P.H.), the National Hospital for Neurology and Neurosurgery, University College London Hospitals; and Department of Neurodegenerative Disease (S.B.), UCL Queen Square Institute of Neurology, London, United Kingdom.

## Abstract

**Objective:**

To characterize the phenotype in individuals with *OPA3*-related autosomal dominant optic atrophy and cataract (ADOAC) and peripheral neuropathy (PN).

**Methods:**

Two probands with multiple affected relatives and one sporadic case were referred for evaluation of a PN. Their phenotype was determined by clinical ± neurophysiological assessment. Neuropathologic examination of sural nerve and skeletal muscle, and ultrastructural analysis of mitochondria in fibroblasts were performed in one case. Exome sequencing was performed in the probands.

**Results:**

The main clinical features in one family (n = 7 affected individuals) and one sporadic case were early-onset cataracts (n = 7), symptoms of gastrointestinal dysmotility (n = 8), and possible/confirmed PN (n = 7). Impaired vision was an early-onset feature in another family (n = 4 affected individuals), in which 3 members had symptoms of gastrointestinal dysmotility and 2 developed PN and cataracts. The less common features among all individuals included symptoms/signs of autonomic dysfunction (n = 3), hearing loss (n = 3), and recurrent pancreatitis (n = 1). In 5 individuals, the neuropathy was axonal and clinically asymptomatic (n = 1), sensory-predominant (n = 2), or motor and sensory (n = 2). In one patient, nerve biopsy revealed a loss of large and small myelinated fibers. In fibroblasts, mitochondria were frequently enlarged with slightly fragmented cristae. The exome sequencing identified *OPA3* variants in all probands: a novel variant (c.23T>C) and the known mutation (c.313C>G) in *OPA3*.

**Conclusions:**

A syndromic form of ADOAC (ADOAC+), in which axonal neuropathy may be a major feature, is described. *OPA3* mutations should be included in the differential diagnosis of complex inherited PN, even in the absence of clinically apparent optic atrophy.

Mutations in *OPA3* (figure 1), encoding a mitochondrial protein likely involved in the regulation of mitochondrial fission,^1–5^ are associated with 2 distinct disorders that share the common feature of bilateral optic atrophy (OA; table e-1, links.lww.com/NXG/A146). Recessive, loss-of-function mutations cause 3-methylglutaconic aciduria type III (MGA3 [MIM 258501]), also known as Costeff syndrome, a rare disorder that is clinically characterized by OA and extrapyramidal dysfunction with onset in the first decade of life, subsequent development of spastic paraparesis and cerebellar signs, and increased urinary excretion of 3-methylglutaconic and 3-methylglutaric acids.^6–13^ Dominant mutations, in contrast, lead to OA that typically presents within the first 2 decades of life and is often associated with cataracts with variable age at onset (autosomal dominant OA and cataract; ADOAC [MIM 165300]).^14–18^ Additional features such as hearing loss, cerebellar and extrapyramidal signs, and dysautonomic or gastrointestinal symptoms have been reported less consistently in patients with ADOAC (table e-2). Symptoms or signs suggestive of peripheral neuropathy (PN) have been described to date in 4 individuals with dominant *OPA3* mutations,^14,15,18^ although it was confirmed by nerve conduction studies (NCSs) in only one of them.^18^

**Figure 1:**
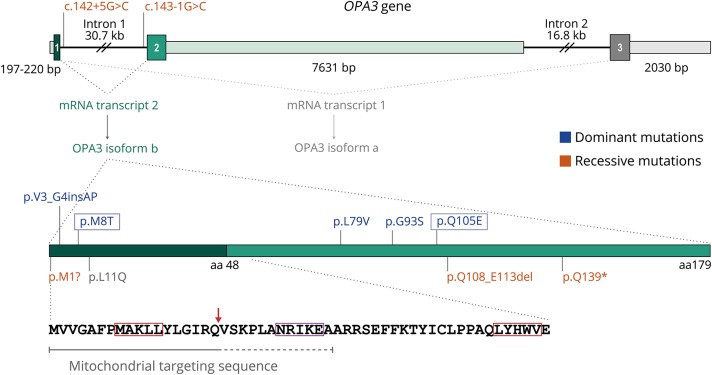
Schematic of the *OPA3* gene and OPA3 protein isoform b The *OPA3* gene (NCBI RefSeq NG_013332.1; top figure), located in chromosome 19q13.32, contains 3 exons (1, 2, and 3; boxes containing exon numbers) and spans 57.4 kb. The coding regions of exons 2 and 3 and their exon-intron boundaries are highly similar and may have originated by segmental duplication.^3^ *OPA3* exons are alternatively spliced to generate 2 mRNA transcripts: transcript variant 2 (exon 1 plus exon 2 [NM_025136.3]) and transcript variant 1 (exon 1 plus exon 3 [NM_001017989.2]). Transcript variant 2 seems to be the predominant transcript in most tissues and encodes a 179 amino acid (AA) protein (OPA3 isoform b [NP_079412.1]; bottom figure). Transcript variant 1 encodes a 180 AA protein (OPA3 isoform a [NP_001017989]; not shown). OPA3 isoform b amino acids 1–48 are encoded by exon 1 (dark green) and amino acids 48–179 are encoded by exon 2 (light green). Its N-terminal region contains a putative mitochondrial targeting sequence in AAs 1–18 (as predicted by *in silico* analysis with MitoProt and TargetP) or 1–30 (as indicated by functional studies with deletion mutants). *In silico* analysis with MitoFates predicts a mitochondrial processing peptidase cleavage site (red arrow) and 2 TOM20 recognition motifs (AAs 8–12 and 48–52; red boxes). An additional mitochondrial sorting/cleavage signal at position 25–29 (purple box) has been proposed by some authors.^3,8^ The localizations of reported dominant and recessive mutations are shown in the figure (one-letter AA abbreviations are used for simplicity; blue boxes indicate mutations reported in the present study). p.Leu11Gln (p.L11Q) was homozygous in 2 siblings with optic atrophy, extrapyramidal signs including dystonia, and pyramidal signs or ataxia, and it was heterozygous in their mother with later-onset dystonia.^30^ No pathogenic mutations have been described in AAs 48-180 of OPA3 isoform a encoded by exon 3 (not shown).

Here, we describe 2 families and one sporadic case with a syndromic form of ADOAC in which PN was a major clinical feature. This report broadens the clinical and genetic spectrum of ADOAC and indicates that *OPA3* should be included in the differential diagnosis of the complex inherited PN^19^ even in the absence of clinically apparent OA.

## Methods

### Patients

The 3 probands were identified at the outpatient clinics of the National Hospital for Neurology and Neurosurgery, London, UK, and the Charcot-Marie-Tooth disease (CMT) Center of Excellence at the University of Pennsylvania, Philadelphia, PA. These probands were recruited into local research protocols to determine the genetic etiology in patients with inherited neuropathies using next-generation sequencing. Diagnostic laboratory tests, neurophysiological studies, MRI scans, and tissue biopsies were performed and analyzed using standard protocols.

### Sequencing

DNA samples were extracted from peripheral blood leukocytes and skeletal muscle biopsy specimens using commercial kits. Exome sequencing was performed after target capture using the Illumina TruSeq Exome, Agilent SureSelect Focused Exome, or Agilent SureSelect Human All Exon kit. The Illumina HiSeq2000 or HiSeq2500 instruments were used to produce 100 bp paired-end sequence reads. The following software tools were used to align sequence reads to the human genome assembly 19 (GRCh37) and to call and annotate variants: Novocraft NovoAlign, Burrows-Wheeler Aligner, Picard, Genome Analysis Toolkit, SAMtools, and ANNOVAR.^20^ After filtering, candidate variants were evaluated *in silico* to predict their effects, and validation and cosegregation analysis of *OPA3* variants in families A, B, and C were performed by Sanger sequencing (see supplementary material for details, links.lww.com/NXG/A146).

Mitochondrial DNA (mtDNA) was assessed for large-scale rearrangements using long-range PCR and Southern blot of total genomic DNA extracted from skeletal muscle. The entire mtDNA sequence was analyzed with Affymetrix GeneChip Human Mitochondrial Resequencing Array 2.0 as described elsewhere.^21^

### Cell imaging

Fibroblast cell lines were established from skin biopsies of proband AII-2 and a healthy age- and sex-matched control using standard methods. Cells were grown in high glucose Dulbecco's modified Eagle’s medium, supplemented with 10% fetal bovine serum, penicillin/streptomycin, and 0.05 mg/mL uridine. For electron microscopy (EM), fibroblasts were fixed in 3% glutaraldehyde in 0.1 M sodium cacodylate buffer and 5 mM CaCl_2_ overnight, then treated with 1% osmium tetroxide for 3 hours at 4°C and embedded in Araldite CY-212 resin. Ultrathin sections (70 μm) were stained with lead citrate and uranyl acetate. Images were taken on a Philips CM10 transmission electron microscope fitted with a MegaView G3 camera and RADIUS Software (Olympus). Mitochondria morphology in fibroblasts was measured blind to disease status.

### Standard protocol approvals, registrations, and patient consents

Research protocols were approved by local institutional review board and/or ethics committee. Patients gave written informed consent to participate.

### Data availability

Anonymized data not published within this article will be made available by request from any qualified investigator.

## Results

### Families and overall phenotype

**Figure 2:**
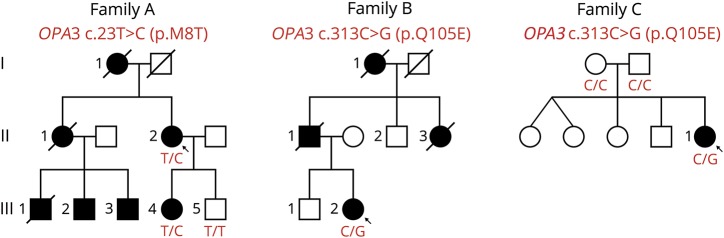
Pedigrees of families and segregation analysis of variants c.23T>C (p.Met8Thr) and c.313C>G (p.Gln105Glu) in *OPA3* (NM_025136.3) *OPA3* variants found in each family and the genotype of tested individuals are indicated in red. Arrow = probands.

Three unrelated probands were referred to our PN clinics for evaluation. Probands AII-2 and BIII-2 had affected relatives of 3 generations based on history (AI-1, AII-1, AIII-2, AIII-3, AIII-4, BI-1, and BII-3) or examination (AIII-1 and BII-1), while proband CII-1 was a sporadic case (figure 2). The clinical information of all affected individuals is summarized in table 1, and their clinical description is provided in the supplementary material (links.lww.com/NXG/A146).

**Table 1:**
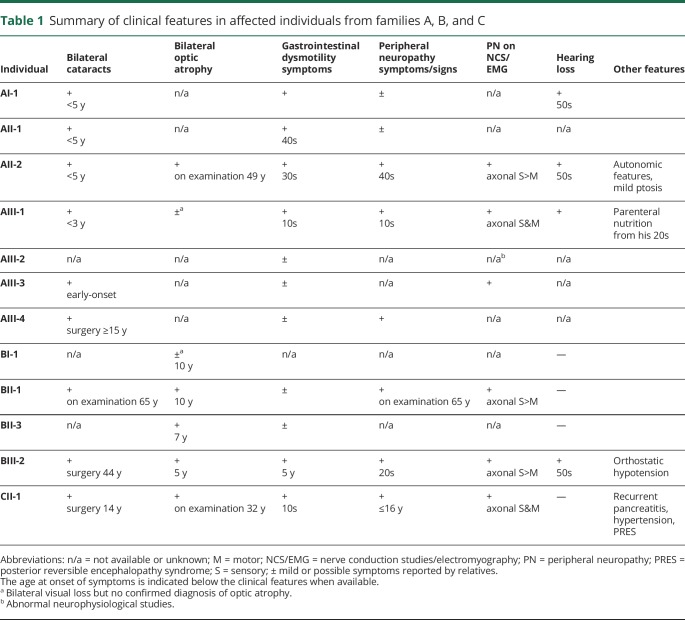
Summary of clinical features in affected individuals from families A, B, and C

#### Family A

**Figure 3:**
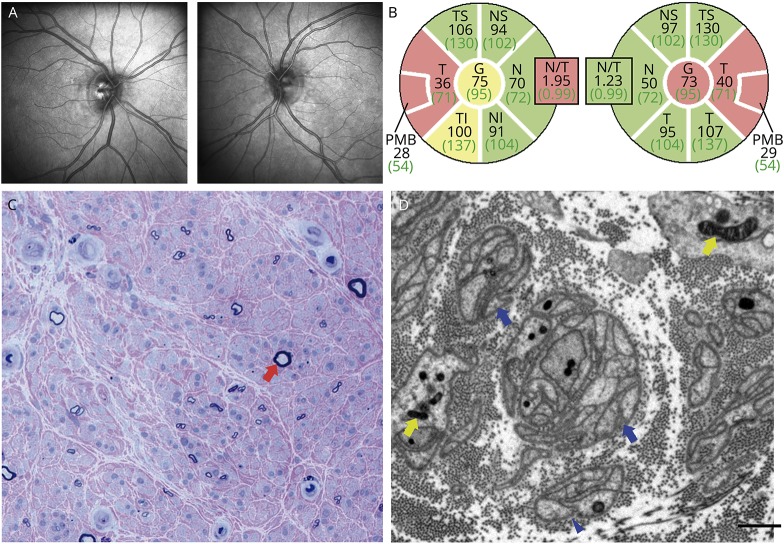
Bilateral optic atrophy and sural nerve biopsy of patient AII-2 (A) Red-free photographs of the optic discs of patient AII-2 show mild temporal pallor of both optic discs. (B) Optical coherence tomography measurements of the retinal nerve fiber layer thickness around both optic discs of patient AII-2 confirm significant thinning in the temporal quadrants consistent with an optic neuropathy. (D) Semi-thin resin section of the sural nerve, stained with methylene blue azure-basic fuchsin, shows a fascicle with severe loss of large (red arrowhead) and small myelinated fibers with no apparent active axonal degeneration and minimal regeneration (scale bar = 25 μm). (E) Electron microscopy shows frequent denervated Schwann cell profiles and bands of Büngner (blue arrowheads) in keeping with widespread fiber loss. The mitochondria (yellow arrowheads) show no apparent pathology (scale bar = 1 μm).

The phenotype in the affected members of family A (n = 7) was mostly characterized by early-onset cataracts, gastrointestinal dysmotility symptoms, and PN (based on symptoms/signs or neurophysiological studies), although the data on these features were incomplete for 3 individuals. Reduced visual acuity was first detected in proband AII-2 during the evaluation of her PN at age 49, and OA was subsequently confirmed (figure 3, A and B). A reduced visual acuity was also observed in AIII-1, but we had no confirmation of an underlying OA. Impaired vision was not reported for other relatives. Intrafamilial phenotypic variability was evident, with patient AIII-1 having more severe symptoms than his siblings, and an earlier and more severe presentation than AII-2.

#### Family B

Early-onset, bilateral visual impairment leading to severely reduced visual acuities by the age of 30–40 was the initial clinical feature in all affected members of family B (n = 4; originally reported in 1996^22^). Three of them had a diagnosis of OA and reported symptoms of gastrointestinal dysmotility. PN and cataracts were detected in proband BIII-2 and her father BII-1 on follow-up examinations in their adulthood.

#### Family C

Proband CII-1 was the only affected individual in this family. Her phenotype was similar to that of family A: early-onset cataracts, gastrointestinal dysmotility symptoms, and PN. In addition, she had recurrent episodes of pancreatitis from age 11. Bilateral OA was detected by reverse phenotyping: after a genetic diagnosis of her PN was achieved, by optical coherence tomography.

### Peripheral neuropathy

Nine of the 12 affected individuals from the 3 families had a possible or confirmed PN. It was considered possible in 4 individuals (AI-4, AIII-2, AIII-3, and AIII-4) based on the history provided by relatives. In 5 patients (AII-2, AIII-1, BII-1, BIII-2, and CII-1), we confirmed the diagnosis based on clinical and neurophysiological assessment (see supplementary material for an extended description, links.lww.com/NXG/A146). Detailed results of NCS/EMG were available in 4 cases (table e-3).

#### Asymptomatic neuropathy

In patient BII-1, the PN was asymptomatic and detected on examination at age 65. NCS were consistent with an axonal motor and sensory neuropathy.

#### Sensory-predominant neuropathy

Probands AII-2 and BIII-2 had a slowly progressive, predominantly sensory neuropathy. AII-2 developed sensory symptoms in her feet in her late 40s and was referred to us for the evaluation of suspected PN at age 49. BIII-2 developed sensory symptoms in her feet in his 20s; her PN was confirmed in her early 30s, but she was referred again to us at age 52 because of symptom worsening. Both patients described reduced sensation restricted to the lower limbs. On serial examinations, we observed distal sensory loss in the upper and lower limbs and signs of mild motor involvement. In both cases, NCS revealed a length-dependent axonal sensory neuropathy. NCS/EMG signs of distal motor involvement were detected only in proband BIII-2.

#### Motor and sensory neuropathy

Proband CII-1 and patient AIII-1 had a moderate-to-severe progressive motor and sensory neuropathy with onset in their childhood or teens. Proband CII-1 had frequent ankle sprains as a child and was referred to us at age 16 when a PN was suspected during physical therapy for a foot fracture. Patient AIII-1 had pes cavus and limb weakness since his teens and had been diagnosed with inherited neuropathy in his early 20s; he was examined by us at age 31 as part of the family evaluation. Both patients exhibited motor and sensory deficits in upper and lower limbs on examination. Proband CII-1 had 4 neurophysiological studies performed between ages 16 and 32, which revealed a severe motor and sensory axonal neuropathy. In patient AIII-3, a previous neurophysiological study at age 29 confirmed the same diagnosis.

### Other clinical features

Symptoms of gastrointestinal dysmotility of variable severity were reported for most individuals. In the best documented cases, symptoms included intermittent constipation (AII-2); episodes of nausea, vomiting, and abdominal pain (CII-1); gastroparesis and episodes of intestinal pseudo-obstruction (BIII-2); and intestinal pseudo-obstruction requiring parenteral nutrition (AIII-1). Patient BIII-2 required emergency surgery for intestinal intussusception in her 50s, and patient AII-1 was also reported to require surgery for her intestinal motility problems.

All probands had features suggestive of autonomic nervous system dysfunction, including orthostatic tachycardia (AII-2), postural hypotension (BIII-2), and episodes of hypertension in the context of gastrointestinal symptoms that, in one occasion, lead to a posterior reversible encephalopathy syndrome (CII-1).

Patient AIII-1 developed hearing loss concurrently to other symptoms. Proband AII-2 complained of mild hearing loss in her 50s, and a bilateral auditory neuropathy was confirmed in her 60s. Patient AI-1 and proband BIII-2 developed hearing loss in their 50s. The less common clinical features are shown in table 1.

### Neuropathology

Patient AII-2 underwent sural nerve and quadriceps muscle biopsy at age 57. Nerve biopsy showed a severe loss of myelinated fibers that was not selective for large or small fibers, with no apparent active axonal degeneration and minimal regeneration, and no evidence of demyelination (figure 3, C and D). Muscle biopsy revealed occasional angular atrophic fibers, 2 cytochrome oxidase-negative fibers, and one ragged-red fiber. The activity of mitochondrial electron transport chain (ETC) complexes I, II + III, and IV was within control ranges, and screening for single/multiple deletions and point mutations of mtDNA in muscle were negative.

### EM of mitochondria

**Figure 4:**
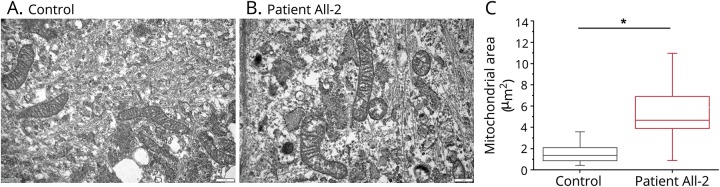
Ultrastructural examination of mitochondria in cultured skin fibroblasts (A) Representative electron micrographs showing the ultrastructure of mitochondria in control and patient AII-2 fibroblasts (scale bar = 1 μm). (B) Patient AII-2 mitochondria (n = 94) display a significant increase in area as compared to control mitochondria (n = 123). (C) Data are presented as box plots illustrating 80% of the data distribution; 10th, 25th, median, 75th, and 90th percentiles are shown for these box plots. *p < 0.0005 (Mann-Whitney *U* test).

Ultrastructural examination of mitochondria with EM was performed in the cultured fibroblasts from proband AII-2 and a healthy control. On EM images, we frequently observed abnormally enlarged mitochondria coupled with slightly fragmented mitochondrial cristae and a concomitant reduction in the area of mitochondrial cristae (figure 4).

### Genetic studies

In probands AII-2, BIII-2, and CII-1, analysis of common genes associated with inherited neuropathy and/or OA yielded negative results. Exome sequencing was then performed on these patients as 3 independent studies. The analysis focused on nonsynonymous, splice-site, and coding indel variants with minor allele frequency <0.1% in the Exome Aggregation Consortium data set, located in genes known to cause inherited neuropathy and/or OA (table e-4, links.lww.com/NXG/A146).

In proband AII-2, we identified the heterozygous missense variant c.23T>C in exon 1 of *OPA3* (table e-5, links.lww.com/NXG/A146). Sanger sequencing detected the mutation in the proband and her affected daughter (AIII-4) but not in the unaffected son (AIII-5), who was homozygous for the wild-type allele (figure 2). c.23T>C is a novel variant absent from public databases and from an in-house control exome database. It affects an evolutionarily conserved nucleotide and is predicted to be deleterious by several bioinformatics tools (table e-6). c.23T>C leads to the substitution of nonpolar, hydrophobic methionine for polar threonine at amino acid (AA) position 8 of OPA3 (p.Met8Thr). This position lies within the N-terminal mitochondrial targeting sequence (MTS) predicted by MitoProt and TargetP software tools (AA 1-18) or by functional studies with deletion mutants (AA 1-30),^2^ in which another dominant mutation has been identified (figure 1).^16^ *In silico* analysis with MitoFates suggests that methionine 8 would be the first of 5 AA of a putative consensus motif recognized by the mitochondrial import receptor subunit TOM20 (figure 1) and that p.Met8Thr would eliminate this recognition motif. The functional significance of hydrophobic AA at position 8 of OPA3 is supported by multiple sequence alignment of OPA3 homologs, which shows conservation of methionine and isoleucine at that position from human to zebra fish (figure e-1). Based on these data, and the previous association of dominant *OPA3* mutations with OA, cataracts, and PN, we considered c.23T>C as the most plausible genetic etiology in family A.

In probands BIII-2 and CII-1, we identified the heterozygous missense mutation c.313C>G (p.Gln105Glu) in exon 2 of *OPA3*. The mutation was validated by Sanger sequencing in both cases. In family B, DNA samples from relatives were not available for cosegregation analysis. In family C, the mutation was not found in either parent, confirming that it had occurred de novo in the proband (figure 2). c.313C>G is not reported in public databases but is the most frequent mutation reported in families with dominant *OPA3*-related OA, sometimes in association with additional features (tables e-1 and e-2, links.lww.com/NXG/A146).^14,16,17^ This variant was therefore classified as likely pathogenic.

## Discussion

Here, we describe the members from 3 families in which a novel missense variant (c.23T>C, p.Met8Thr) and a known mutation (c.313C>G, p.Gln105Glu) in *OPA3* were the most likely underlying cause of a complex phenotype consisting of OA, cataracts, gastrointestinal dysmotility, axonal neuropathy, and possibly autonomic dysfunction and hearing loss. This observation, together with 2 former reports of similar phenotypes associated with the missense substitutions p.Leu79Val and p.Gln105Glu,^15,18^ demonstrates that the dominant *OPA3* mutations can cause syndromic forms of ADOAC (ADOAC “plus”).

We also confirm that an axonal neuropathy may be a major clinical feature of ADOAC/+. In 4 patients from our series, the PN was indeed a major cause of disability or was severe enough to motivate the referral to our centers. In 5 patients with a well-documented axonal neuropathy, we observed 3 clinical presentations: asymptomatic, sensory-predominant, and motor and sensory. However, given the differences in the age of symptoms onset and overall disease severity between patients, and the later development of motor signs in patients with sensory-predominant forms, it is possible that the different PN presentations may simply reflect a variable expressivity of the same pathologic process rather than distinct PN subtypes. A sensory ganglionopathy seemed unlikely in patients with sensory-predominant forms, in whom the PN was length-dependent and symmetrical, with almost normal joint position sense and no significant limb ataxia.

Despite the limited sample size, our report also suggests a higher prevalence of PN among patients with ADOAC/+ than previously recognized. Possible reasons for this are that the PN may be asymptomatic and/or develop several decades after the onset of the ophthalmologic problems. Therefore, long-term follow-up with neurologic evaluations may be necessary to confirm or exclude a PN in these patients. On the other hand, data from this and previous studies indicate that the dominant *OPA3* mutations may exhibit considerable phenotypic variability, so it is also possible that the families or individuals with the same mutation may or may not develop additional features such as PN. This point should be clarified in future studies encompassing larger sample sizes.

Symptoms of gastrointestinal dysmotility were reported for most affected individuals from the present series, which so far has only been described in 3 patients with ADOAC/+.^15,16,18^ Results from gastrointestinal investigations were not available for review, and thus, we could not confirm the underlying pathophysiology. Its coexistence with cardiovascular dysautonomic features and/or PN in cases from the present and previous studies raises the possibility of a myenteric plexus or extra-intestinal autonomic neuropathy, although we cannot exclude other causes such as an enteric myopathy. Of note, gastrointestinal dysmotility is a well-recognized manifestation of certain mitochondrial disorders, for which both neuropathic and myopathic mechanisms have been proposed.^22^

The precise function of the OPA3 protein and the molecular mechanisms by which heterozygous missense mutations in *OPA3* cause ADOAC/+ are unknown. OPA3 predominantly localizes to the mitochondria^2–4^ but its intramitochondrial topology is still discussed: the mouse homolog of OPA3 copurifies with the inner mitochondrial membrane,^1^ but the studies on subcellular fractions of HeLa cells suggest that OPA3 may be anchored to the mitochondrial outer membrane with the C-terminus exposed to the cytoplasm.^2^ Overexpression of OPA3 induces mitochondrial fragmentation, whereas downregulation leads to more elongated and tubular mitochondria.^2,5^ These data support that OPA3 is a mitochondrial membrane protein implicated in the regulation of mitochondrial fission and morphology.

Heterozygous carriers of the recessive *OPA3* mutation c.143-1G>C, which abolishes mRNA expression, are asymptomatic.^8,23^ This suggests that dominant missense mutations have a dominant-negative effect or result in a gain-of-function rather than haploinsufficiency. Fibroblasts from ADOAC patients with the p.Val3_Gly4insAlaPro mutation, located in the N-terminal MTS of OPA3, showed increased fragmentation of the mitochondrial network,^16^ which mimics *OPA3* overexpression and gives support to the gain-of-function hypothesis. Similar findings were observed in HeLa cells transfected with an OPA3 mutant carrying the p.Gly93Ser mutation, located in the hydrophobic region of OPA3 (AA 83-120) that is required for mitochondrial fragmentation.^2^ However, no mitochondrial network abnormalities were detected in fibroblasts from one patient with the same mutation.^14^ Therefore, it is possible that dominant mutations affecting different regions of OPA3 may exert different deleterious effects.

Studies in a zebra fish model of MGA3 indicate that OPA3 does not have a direct role in mitochondrial ETC function,^24^ and consistent with this, we found the normal activities of ETC complexes in skeletal muscle from patient AII-2. We did not detect large-scale mtDNA rearrangements in muscle, which argues against a role of OPA3 in mtDNA maintenance. However, EM analysis of mitochondria in fibroblasts from patient AII-2 revealed frequently enlarged mitochondria with abnormal cristae. Subtle alterations to the morphology of mitochondrial cristae have also been identified in the retinal tissues of a mouse model of MGA3 caused by the homozygous missense mutation p.Leu122Pro.^4^ Therefore, OPA3 function might be important for the structural integrity of mitochondrial cristae, and altered mitochondrial cristae architecture could be an additional mechanism involved in the pathogenesis *OPA3*-related disease.

Based on the findings from the present study, some parallels can be drawn between *OPA3*- and *OPA1*-related diseases. Mutations affecting OPA1, a dynamin-related guanosine triphosphatase involved in mitochondrial dynamics, represent a major cause of dominant OA (DOA), and ∼20% of patients may present a DOA “plus” phenotype, including axonal neuropathy.^25^ Biallelic mutations in *OPA1* can also cause Behr syndrome,^26–28^ a severe, early-onset neuro-ophthalmologic syndrome that is clinically similar to MGA3. Of interest, the loss of OPA1 causes disruption of mitochondrial cristae structures.^29^ Overall, these data may indicate shared pathogenic mechanisms between *OPA3* and *OPA1* defects that warrant further research.

## Glossary

AA: amino acid
ADOAC: autosomal dominant optic atrophy and cataracts
DOA: dominant optic atrophy
EM: electron microscope
ETC: electron transport chain
MGA3: 3-methylglutaconic aciduria type III (Costeff syndrome)
MTS: mitochondrial targeting sequence
NCS: nerve conduction study
OA: optic atrophy
OPA3: Optic atrophy 3 protein
PN: peripheral neuropathy
